# Isolation and Genomic Characteristics of the First Bovine Viral Diarrhea Virus Subgenotype 2b Isolate from Buffalo in Guangxi Province, China

**DOI:** 10.3390/microorganisms14081845

**Published:** 2026-08-19

**Authors:** Shuhong Zhong, Shaomin Qin, Shiwen Feng, Cuilan Wu, Lan Jia, Xiongbiao Xuan, Huili He, Hao Peng, Shuai Hu, Jinfeng Liu, Jun Lin, Jun Li

**Affiliations:** 1 Guangxi Key Laboratory of Veterinary Biotechnology, Guangxi Veterinary Research Institute, Nanning 530001, China; zhongshuhong11@163.com (S.Z.); 0134130120@163.com (S.Q.); fengshw1205@163.com (S.F.); cuilanwu@163.com (C.W.); xuanxiongbiao@163.com (X.X.); hhl19870820@163.com (H.H.); hpeng2006@163.com (H.P.); benlaolao0000@163.com (S.H.); fjinliu@163.com (J.L.); 2 Key Laboratory of China (Guangxi)-ASEAN Cross-Border Animal Disease Prevention and Control, Ministry of Agriculture and Rural Affairs of China, Nanning 530001, China; 3 College of Animal Science and Technology, Guangxi University, Nanning 530005, China; jialan0830@163.com

**Keywords:** bovine viral diarrhea virus type 2b, buffalo, phylogenesis, recombination, B-cell epitope, glycan shielding, China

## Abstract

(1) Background: Bovine viral diarrhea virus (BVDV) is an economically important pathogen affecting cattle worldwide. The genetic diversity of BVDV-2 in buffalo remains poorly understood. This study aimed to elucidate the genomic and antigenic features of GX24, the first BVDV-2b isolate identified from dairy buffalo in China. (2) Methods: The virus was isolated from a rectal swab through three blind passages in MDBK cells and identified through immunofluorescence and RT-PCR. The near-complete genome was sequenced using the Illumina platform and subjected to phylogenetic and recombination analysis. B-cell epitopes and glycosylation sites were predicted using BepiPred-3.0, Epitope1D, DiscoTope-3.0, NetNGlyc-1.0, and NetO-Glyc-4.0. (3) Results: The 12,267-nt GX24 genome (GenBank: PX682047) was classified as BVDV-2b. Recombination analysis detected a putative recombination signal within the NS5A gene involving Chinese BVDV-2b and Italian BVDV-2a strains. Integrative analyses predicted conserved linear and conformational epitope clusters in the N- and C-terminal regions of the E2 protein. Spatial analysis indicated that several epitope residues may be masked by glycan shielding. (4) Conclusions: This study provides the first genomic evidence of a BVDV-2b isolate from dairy buffalo in China, suggesting that BVDV-2b may be present in this population. The putative recombination signal and antigenic characteristics offer valuable insights for the development of diagnostics and vaccine design in the future.

## 1. Introduction

As a major causative pathogen of bovine respiratory disease complex (BRDC) and neonatal calf diarrhea (NCD), bovine viral diarrhea viruses (BVDV) is an economically significant infectious agent affecting cattle worldwide [1,2,3]. Clinical manifestations of BVDV infection include immunosuppression, growth retardation, reproductive disorders, and the generation of persistently infected (PI) animals [4]. PI animals serve as the primary natural reservoir of BVDV [5]. The hosts range of BVDV extends to nearly all members of the order *Artiodactyla*, including swine, goats, and deer. Notably, the European rabbit (*Oryctolagus cuniculus*) and the European hare (*Lepus europaeus*) are also susceptible to BVDV infection; however, the epidemiological significance of these *Lagomorph* species remains unclear [6].

BVDVs (genus: *Pestivirus;* family: *Flaviviridae*) consists of a single-stranded, positive-sense RNA molecule approximately 11.3–13.0 kb in length, containing a single large open reading frame (ORF). The ORF encodes four structural proteins (Cap, E^rns^, E1 and E2) and eight non-structural proteins (N^pro^, p7, NS2, NS3, NS4A, NS4B, NS5A and NS5B), flanked by untranslated regions (UTRs) [7]. BVDV serves as an umbrella term encompassing three recognized *Pestivirus* species, BVDV-1 (*P. bovis*), BVDV-2 (*P. tauri*), and HoBi-like virus or BVDV-3 (*P. brazilense*) [7,8,9]. Based on the phylogenetic analyses of complete or near-complete genomes (CNCGs) or partial genomic regions (e.g., 5′ UTR, N^pro^, E2, NS4A and NS5A), BVDV-1 is currently classified into 25 subgenotypes (1a–1x), BVDV-2 into five subgenotypes (2a–2e), and BVDV-3 into five subgenotypes (3a–3e) [9,10,11,12,13,14,15].

In China, BVDV-1 was first reported in the 1980s, followed by BVDV-2 in 2009, and BVDV-3 in 2016 [16,17,18,19]. Over the past two decades, increasing demand for beef and dairy products has intensified nationwide cattle transportation, facilitating the spread of BVDV across provinces in China [20]. The virus has been detected in multiple animal species, including cattle, camels, yaks, water buffalo, sheep, goats, and swine [16]. Epidemiological surveys have demonstrated that BVDV infection is widespread throughout China, although prevalence varies considerably according to region, host species, and detection method. At the herd level, seropositivity reached 93.33% (28/30) among large-scale dairy farms across seven western provinces during 2019–2022 and 77.8% (28/36) among dairy herds in five eastern provinces in 2018 [21,22]. At the individual level, seroprevalence was highest in dairy cattle (89.49%, 298/333), followed by beef cattle (63.27%, 248/392), yaks (45.38%, 236/520), and water buffalo (14.18%, 19/134) across eight provinces representing northern, central, eastern, western, and southern China during 2010–2013 [23]. Among cross-regionally transported feeder cattle, the overall seroprevalence was 78.85% (5986/7592), with significantly higher seroprevalence in southwestern receiving regions (Sichuan Province, 89.59%; Chongqing Municipality, 88.15%) than in northern production regions (Hebei Province, 76.31%; Inner Mongolia Province, 76.08%; Liaoning Province, 76.49%). Furthermore, seroprevalence increased from 73.97% in 2022 to 85.04% in 2024 [24]. Antigen and RNA detection further indicated active infection, although at lower prevalence than serological evidence. Antigen-positive rates were 1.39% (14/1010) across the same eight provinces [23] and 1.86% (7/377) among dairy herds in eastern China [22]; RT-PCR analysis detected viral RNA in 22.64% (146/645) of samples collected from eight provinces [23], 7.01% (467/6651) of cross-regionally transported cattle [24], and 2.03% (6/295) of diarrheic calves in Guangdong Province during 2021–2023 [25]. China currently exhibits one of the greatest genetic diversities of BVDV in Asia, with 11 BVDV-1, two BVDV-2, and two BVDV-3 subgenotypes identified to date [9,15]. In China, the predominant subgenotypes circulating in cattle are BVDV-1b, BVDV-1m, and BVDV-1q [23]; whereas, the prevalence of BVDV-2 has increased in recent years [26,27]. Of the four globally recognized BVDV-2 subgenotypes (2a–2d), only BVDV-2a and BVDV-2b have been reported in China. BVDV-2a was first isolated from cattle in Xinjiang Province (strain XJ-04) [18], followed by the identification of strain SH-28 from pigs in Shanghai Municipality [28]. In addition, both NCP (non-cytopathic) and CP (cytopathic) BVDV-2 strains have also been isolated from large-scale dairy farms in western China [21]. In China, BVDV-2b was first identified in cattle in 2013 (strain SD1301, Jilin Province) [29] and has subsequently been reported in Henan Province (strain BVDV-HEN01) [30], Yunnan Province (strain YNJG) [31], and Inner Mongolia Province (strain BVDVNM21, a highly virulent NCP isolate) [32]. However, before the present study, BVDV-2b had not been reported in dairy buffalo in China. Comprehensive genomic characterization of circulating BVDV strains is essential for understanding viral evolution, guiding control strategies, and supporting the development of effective vaccines and diagnostic tools. In the present, we report the genomic characterization of a BVDV-2b strain isolated from dairy buffalo in China.

## 2. Materials and Methods

### 2.1. Samples, Cells and Virus Isolation

A rectal swab was collected in 2024 from a diarrheic buffalo at a dairy buffalo farm in Guangxi Province, China. The swab was immersed in 2 mL of Minimum Essential Medium (MEM, GIBCO, Thermo Fisher Scientific, Waltham, MA, USA ) and vortexed thoroughly. The resulting suspension was clarified by centrifugation at 5000× *g* for 5 min, and the supernatant was filtered through a 0.22 µm polyvinylidene difluoride (PVDF) membrane. Madin-Darby bovine kidney (MDBK) cells were obtained from the China Center for Type Culture Collection (CCTCC, Wuhan, China) and cultivated in MEM supplemented with 10% fetal bovine serum (FBS, HyClone, GE Healthcare Life Sciences, Logan, UT, USA). The cells were maintained at 37 °C in a humidified atmosphere containing 5% CO_2_. The clarified supernatant was inoculated onto confluent MDBK cells monolayers and incubated for 5 days. The cells were then subjected to three freeze–thaw cycles to release intracellular virus, and the culture was passaged blindly thrice on fresh MDBK cells. The cell cultures were monitored daily for the appearance of cytopathic effects (CPE). In the absence of CPE, viral infection was confirmed through the immunofluorescence assay (IFA) with a BVDV-specific monoclonal antibody (mAb, VMRD, Inc., Pullman, WA,USA) and the reverse transcription polymerase chain reaction (RT-PCR), as previously described [32]. The cell cultures were harvested and negative-stain preparations for transmission electron microscopy (TEM) were made as described previously [33]. The virions were observed and photographed.

### 2.2. Genome Sequencing and Assembly

Total RNA was extracted from the infected cell cultures using VeZol Reagent (Vazyme Biotech Co., Ltd., Nanjing, China) according to the manufacturer’s instructions. Then, the RNA quality was quantified using the Biodropsis BD-1000 (OSTC, Beijing, China). A high-quality RNA sample (Concentration ≥ 50 ng/μL) was used for viral genome sequencing. Genome sequencing and assembly were performed by Sangon Biotech (Shanghai) Co., Ltd. (Shanghai, China). The sequencing library was constructed with an insert size of approximately 350 bp and sequenced on the Illumina HiSeq platform using a paired-end strategy (2 × 150 bp). A total of 76,871,644 raw paired-end reads (~11.5 Gb) were generated. The raw data quality was assessed using FastQC v0.11.2 (https://www.bioinformatics.babraham.ac.uk/projects/fastqc/(accessed on 20 December 2024)), and quality trimming was performed using Trimmomatic v0.36 as per the following steps: (i) removal of adapter sequences (forward: 5′-AGATCGGAAGAGCACACGTCTGAAC-3′; reverse: 5′-AGATCGGAAGAGCGTCGTGTAGGGA-3′); (ii) removal of reads containing ambiguous bases (N); (iii) trimming of low-quality bases (Phred quality score < 20) from both the 5′→3′ and 3′→5′ directions; (iv) sliding-window trimming of trailing low-quality bases (window size: 5 bp, quality threshold: <20); and (v) exclusion of reads and their paired reads < 35 nt [34]. After quality control, 74,739,428 clean reads (~9.3 Gb) were retained. To minimize host genomic background, clean reads were first mapped against an internal viral/plasmid database to extract virus-enriched reads (pre-assembly screening). This step functions as host depletion and read enrichment, analogous to physical viral particle enrichment in wet-lab protocols. The enriched reads were then de novo assembled using SPAdes v3.5.0 without any reference-sequence guidance [35]. Assembled contigs were subjected to gap closure using GapFiller v1.11 and then sequence correction using PrInSeSG v1.0.0 [36,37]. Remapping of the clean reads to the assembled genome yielded a mean depth of 50.91×, with 100% of the nucleotide positions covered at ≥1× and 98.9% (12,127/12,264) covered at ≥10×; no zero-coverage positions were observed, which is consistent with the complete absence of ambiguous bases in the final consensus sequence. The final consensus sequence of 12,267 nt contained zero ambiguous bases (N = 0).

### 2.3. Genome Characteristics, Phylogenetic and Genetic Variability Analysis

Viral genome characteristics were determined by allelic matching against the standard reference sequences XJ-04 (FJ527854) and SD1301 (KJ000672) to annotate gene boundaries and genome organization [18,29].

A total of 96 *Pestivirus* CNCGs retrieved from GenBank, including 25 BVDV-1, 64 BVDV-2, 5 BVDV-3, and 2 other *Pestivirus* members (*P. giraffae*, AF144617; *P. aydinense*, JX428945) as outgroups, were used to construct the phylogenetic tree. The reference sequences were selected as a representative set and did not include all BVDV CNCGs publicly available in GenBank. The selection criteria included: (i) complete or near-complete genome status (≥11 kb); (ii) coverage of all three BVDV genotypes (BVDV-1, -2, and -3), including all subgenotypes for which complete or near-complete genome sequences were publicly available: 17 BVDV-1 subgenotypes (1a–1k, 1m–1o, 1q, 1r, and 1u), 4 BVDV-2 subgenotypes (2a–2c and 2e), and 4 BVDV-3 subgenotypes (3a and 3c–3e); (iii) geographic representation from Asia, Europe, and the Americas, with all publicly available BVDV-2 CNCGs from China (*n* = 17) and from all of China’s neighboring countries with publicly available BVDV-2 genomes (Japan, n = 4; South Korea, n = 2) included to ensure exhaustive regional sampling; and (iv) isolation years spanning 1959–2024 to capture temporal diversity. The background of these reference sequences is listed in Table S1. All 97 sequences were aligned using MAFFT v7.526 [38]. An unrooted maximum-likelihood (ML) phylogenetic tree was constructed using IQ-TREE v3.1.1, with the significance of branching order determined through bootstrap analysis with 1000 replicates [39]. The best-fit substitution model (GTR+F+R5) was identified using ModelFinder implemented in IQ-TREE v3.1.1 based on the Bayesian Information Criterion (BIC). Using the same CNCG alignment, pairwise evolutionary distances were estimated using the Kimura 2-parameter (K2P) method in MEGA v12.1.2. In addition, uncorrected nucleotide p-distances were calculated using MEGA v12.1.2 to determine observed sequence identities [40]. K2P distances were used to describe model-corrected evolutionary divergence; whereas, nucleotide sequence identity was calculated as 100% minus the corresponding p-distance. The phylogenetic tree and the K2P evolutionary distance matrix were visualized and modified using the web application tvBOT v2.6.1 (https://www.chiplot.online/tvbot.html (accessed on 12 April 2026)) [41]. The p-distance matrix was used only to calculate observed nucleotide sequence identities.

### 2.4. Recombination Analysis

Recombination events among the 66 BVDV-2 CNCGs were analyzed using Recombination Detection Program 5 (RDP5) software v5.83 [42]. Putative recombination events were identified using the following seven algorithms: RDP, GENECONV, Bootscan, Maxchi, Chimaera, SiScan, and 3Seq. Bayesian phylogenetic inference was performed using Markov chain Monte Carlo (MCMC) methods with the MrBayes3.2 module implemented in RDP5 [42,43], and the results were visualized using tvBOT v2.6.1 (https://www.chiplot.online/tvbot.html (accessed on 13 April 2026)) [41]. The similarity map of the putative recombinant and its parental isolates was generated using SimPlot++ v1.3 [44].

### 2.5. B-Cell Epitopes Prediction of E2 Protein

Four representative Chinese BVDV-2 E2 amino acid sequences: XJ04 (FJ527854), SD1301 (KJ000672), YNJG2020 (MW168422) and 2b (PP854293), were selected as references based on the subgenotype and year of isolation. Full-length E2 amino acid sequences were submitted to SignalP-6.0 (https://services.healthtech.dtu.dk/services/SignalP-6.0/(accessed on 6 May 2026)) for the prediction of signal peptides and to DeepTMHMM (https://services.healthtech.dtu.dk/services/DeepTMHMM-1.0/ (accessed on 6 May 2026)) for transmembrane topology prediction [45,46]. The predicted signal peptide, transmembrane domain, and intracellular domain were removed, and the remaining ectodomain sequence was applied for B-cell epitope prediction.

Linear B-cell epitopes were predicted using the BepiPred-3.0 method and Epitope1D methods [47,48]. BepiPred-3.0 was accessed via the B Cell Epitope Prediction (Sequence Input) module of the Next-generation IEDB tools (https://nextgen-tools.iedb.org/pipeline?tool=bcell_sequence (accessed on 6 May 2026)) [49]. The putative epitope regions were defined as residues with scores above the default thresholds of 0.151. To validate these predictions, the same sequences were submitted to Epitope1D (https://biosig.lab.uq.edu.au/epitope1d/(accessed on 6 May 2026)) with the default thresholds of 0.5 [48]. Regions containing at least six consecutive amino acids predicted by both methods were extracted as high-probability epitopes. As no experimentally confirmed linear B-cell epitope data for BVDV E2 are currently available in the IEDB database (https://www.iedb.org/ (accessed on 7 May 2026)), a protein-specific length distribution analysis could not be performed. Accordingly, the six-residue cutoff was therefore adopted as an empirical lower bound, supported by the observation that 99% of experimentally confirmed linear epitopes have lengths of at least 6 residues [48]. Shorter overlaps between two independent predictors seemed more likely to reflect algorithmic coincidence rather than genuine epitope regions. This cutoff effectively excluded spurious short overlaps while retaining the biologically plausible epitope candidates. We thereby acknowledged that this is an empirical strategy and that future experimental mapping of BVDV E2 epitopes may enable a more tailored threshold. The use of overlapping predictions from two independent methods follows a consensus-based strategy. For example, BepiPred-3.0 and Epitope1D employ fundamentally different algorithms: BepiPred-3.0 uses ESM-2 protein language model embeddings with a feed-forward neural network [47]; whereas, Epitope1D uses graph-based signatures, antigenicity propensity scales, and organism ontology information [48]. Previous benchmarking studies have demonstrated that individual epitope prediction methods exhibit limited generalization across data sources [48]; whereas, BepiPred-3.0′s performance varies substantially across independent test sets [47,48]. Regions predicted by both methods are therefore less likely to represent false positives, which is consistent with the consensus approach widely adopted in bioinformatics prediction studies.

For conformational B-cell epitope prediction, the ectodomain sequences were submitted to the AlphaFold Server (https://alphafoldserver.com(accessed on 13 May 2026)) for tertiary structure prediction [50]. The resulting mmCIF files were converted to the PDB format using UCSF ChimeraX v1.11.1, and the residues with predicted local distance difference test (pLDDT) scores < 70 were removed so as retain only high-confidence regions [51,52]. Residues with predicted local distance difference test (pLDDT) scores < 70 were removed to retain only the high-confidence regions. Low-confidence residues were identified and removed in UCSF ChimeraX v1.11.1 using the command select & @CA & @@bfactor < 70; select up; delete sel, which selects all Cα atoms with b-factor (pLDDT) values below 70, expands the selection to the entire residue, and deletes the selected residues. Pairwise structural alignments were then performed using the Matchmaker tool in UCSF ChimeraX v1.11.1, which matched 318–321 atom pairs (predominantly Cα atoms) per comparison, with 239–318 pruned atom pairs after iterative outlier removal. The Root-mean-square deviation (RMSD) values were calculated from the pruned atom pairs. An RMSD < 2 Å was interpreted as indicating high structural similarity. Conformational B-cell epitopes were predicted using the B Cell Prediction (Structure Input) module of the IEDB Analysis Resource with the DiscoTope-3.0 method (https://nextgen-tools.iedb.org/pipeline?tool=bcell_structure (accessed on 15 May 2026)), applying the higher-confidence threshold of 1.50 (recall ~30%) [49,53].

The putative N-linked glycosylation sites were predicted using NetNGlyc-1.0 (https://services.healthtech.dtu.dk/services/NetNGlyc-1.0/ (accessed on 20 May 2026)), O-GalNAc (mucin-type) glycosylation sites using NetO-Glyc-4.0 (https://services.healthtech.dtu.dk/services/NetOGlyc-4.0/(accessed on 20 May 2026)), and C-mannosylation sites using NetCGlyc-1.0 (https://services.healthtech.dtu.dk/services/NetCGlyc-1.0/(accessed on 21 May 2026)) [54,55,56]. Predicted epitopes and glycosylation sites were mapped onto the structural models in UCSF ChimeraX v1.11.1. A glycosylation site was considered to shield a conformational epitope if the shortest spatial distance between the glycosylation site and any epitope residue was <10 Å.

## 3. Results

### 3.1. Virus Isolation and Identification

**Figure 1:**
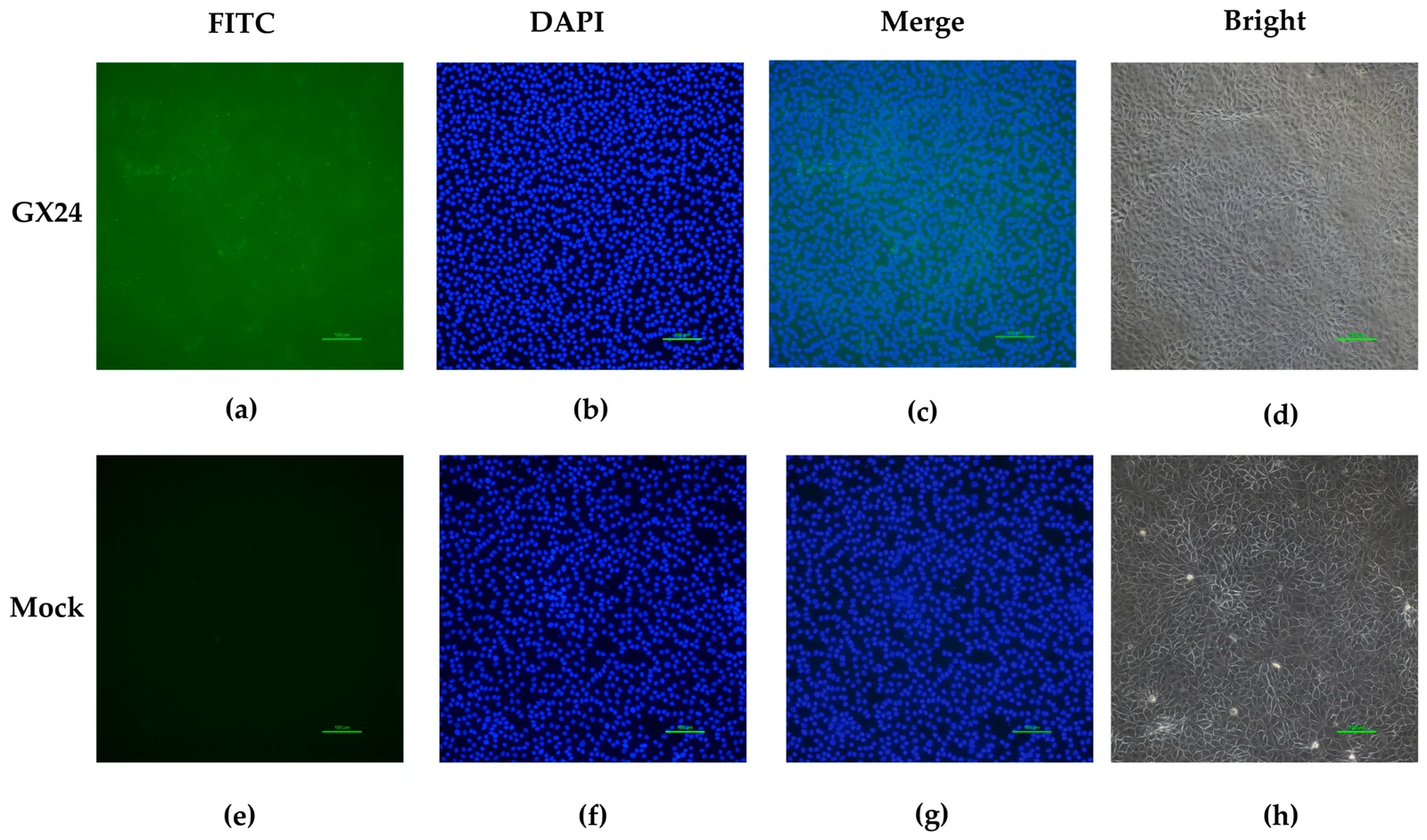
Immunofluorescence assay (IFA) and bright-field microscopy of MDBK cells infected with GX24. (**a**–**d**) GX24-infected MDBK cells: (**a**) FITC channel showing granular virus-specific fluorescence distributed throughout the cytoplasm; (**b**) DAPI nuclear staining; (**c**) merged FITC and DAPI channels; (**d**) bright-field image showing an intact cell monolayer without cytopathic effects. (**e**–**h**) Mock-infected control MDBK cells: (**e**) FITC channel showing no specific fluorescence; (**f**) DAPI nuclear staining; (**g**) merged image; (**h**) bright-field image. Scale bar, 100 µm.

The virus was successfully isolated after three blind passages in MDBK cells. No CPE was observed in the infected cells throughout the isolation process. RT-PCR amplified the expected 283 bp fragment (Figure S1a), confirming the presence of the viral genome. IFA using BVDV-specific mAb revealed distinct granular fluorescence distributed throughout the cytoplasm of infected MDBK cells (Figure 1). A spherical virion of approximately 50nm in diameter, morphologically consistent with members of the genus *Pestivirus,* was observed in negative-stain preparations of the cell culture supernatant (Figure S1b). These findings confirming successful isolation of BVDV, and the isolate was designated GX24.

### 3.2. Genome Characteristics, Phylogenetic Analysis and Genetic Diversity of GX24

The CNCG of GX24 was 12,267 nucleotides (nt) in length and comprised a single ORF of 11694 nt flanked by a partial 5′ UTR of 371 nt and a partial 3′ UTR of 202 nt (Table 1). The genome sequence has been deposited in GenBank under accession number PX682047.

**Figure 2:**
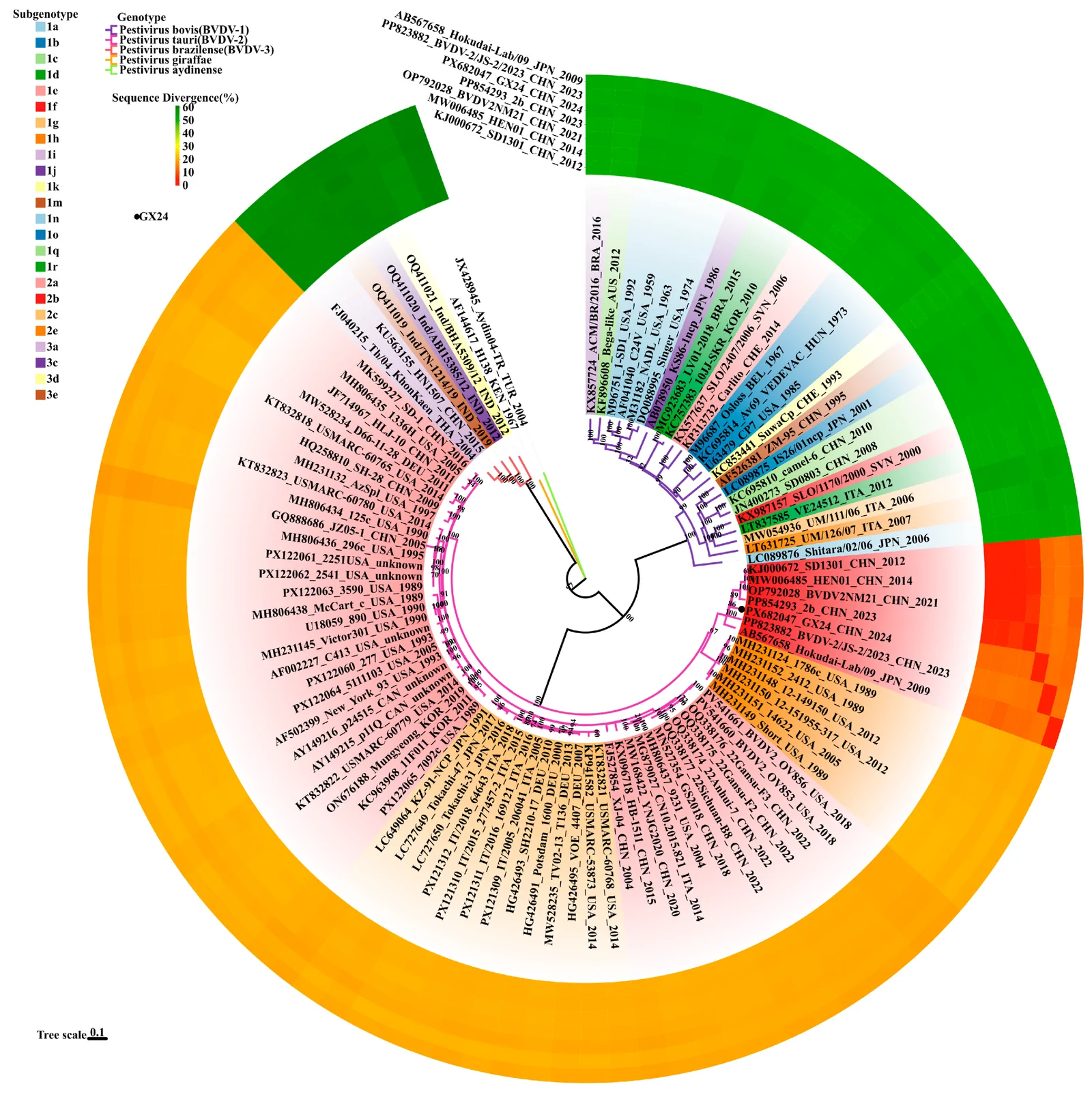
ML phylogenetic tree based on BVDV CNCGs. The tree was reconstructed using IQ-tree v3 under the GTR+F+R5 nucleotide substitution model with 1000 bootstrap replicates. Major BVDV genotypes are indicated by different branch colors; whereas, subgenotypes are distinguished by distinct leaf colors. Each tip is labeled as “GenBank accession number_isolate name_Country code_isolated year”, with country codes following the ISO 3166-1 alpha-3 standard [57]. GX24 is marked by a black dot. The outer ring heatmap illustrates pairwise K2P evolutionary distances between the BVDV-2 isolates and the other isolates included in this study. The color gradient ranges from bright green (0% divergence) to red (60% divergence).

The maximum-likelihood (ML) phylogenetic tree constructed using BVDV CNCGs classified GX24 within the BVDV-2b subgenotype (Figure 2). Pairwise K2P evolutionary distances between GX24 and BVDV-3, BVDV-1, and BVDV-2 isolates ranged from 58% to 60%, 50% to 54%, and 12% to 22%, respectively. The K2P distances between GX24 and other BVDV-2b isolates ranged from 12% to 14%. Because K2P distances represent model-corrected evolutionary distances rather than directly observed mismatch percentages, uncorrected p-distances were additionally calculated in MEGA v12.1.2 to estimate nucleotide sequence identity (Table S2). GX24 shared 67.23–67.82% nucleotide sequence identity with BVDV-3 isolates, 69.72–70.60% identity with BVDV-1 isolates, and 84.32–89.64% identity with other BVDV-2 isolates. Notably, GX24 exhibited the highest nucleotide sequence identity with other BVDV-2b isolates (89.15–89.64%), compared with 84.32–86.07% identity with BVDV-2a isolates, supporting its assignment to BVDV subgenotype 2b.

### 3.3. Recombination Analysis of GX24

**Figure 3:**
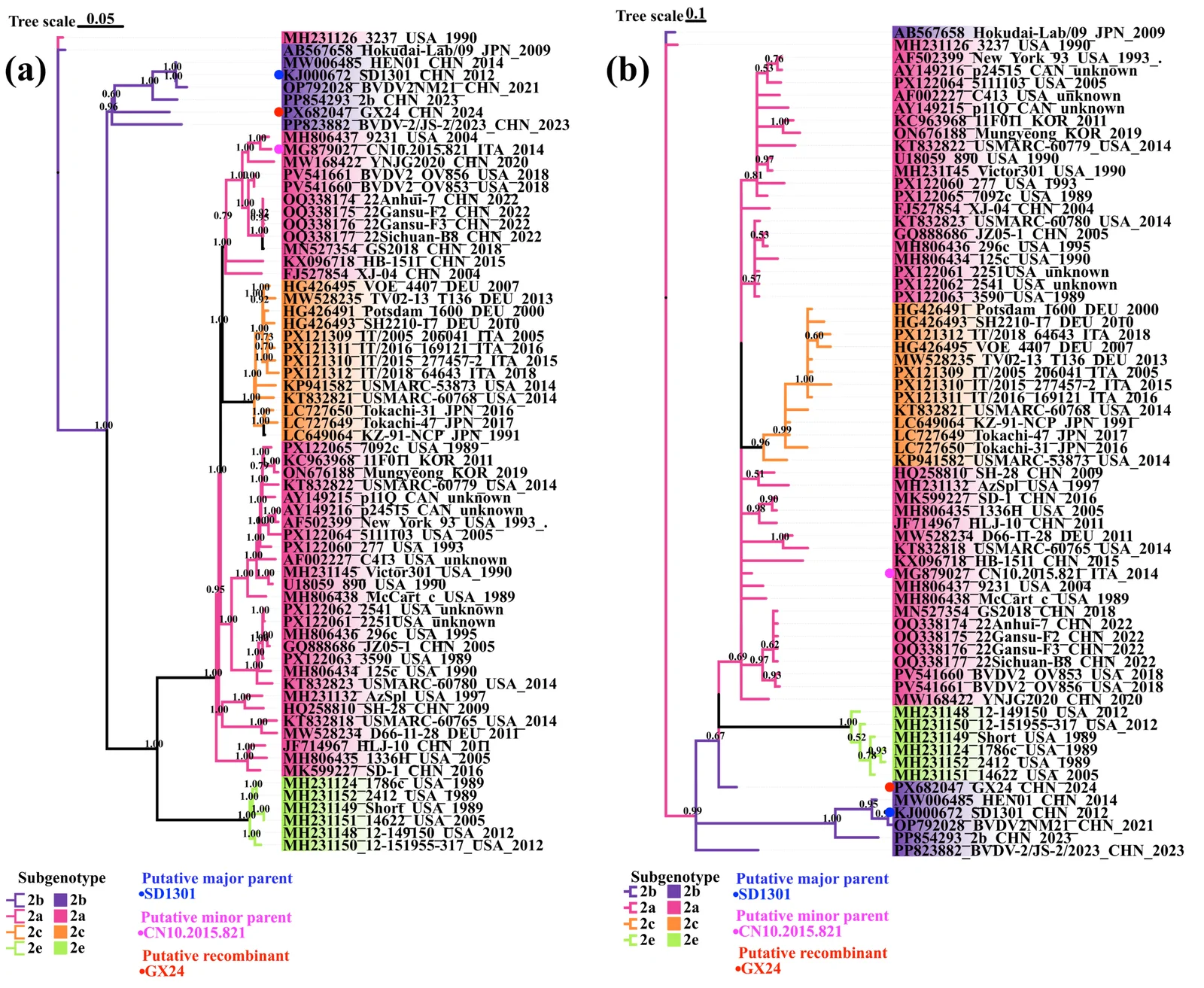
Bayesian phylogenetic trees of BVDV-2 CNCGs. Analyses were performed on datasets excluding the putative recombinant regions of the isolate GX24: (**a**) excluding the region derived from the minor parent; (**b**) excluding the region derived from the major parent. Trees were inferred using the Markov chain Monte Carlo (MCMC) method with the GTR model for 10,000,000 generations using Mrbayes3.2 within the RDP5 software. The subgenotypes are distinguished by leaf colors. Leaves are labeled as “GenBank accession number_isolate name_Country code (ISO 3166-1 alpha-3 code) _isolated year.” The putative recombination (GX24), major parent (SD1301) and minor parent (CN.10.2015.821) are indicated by red, blue, and magenta dots, respectively.

**Figure 4:**
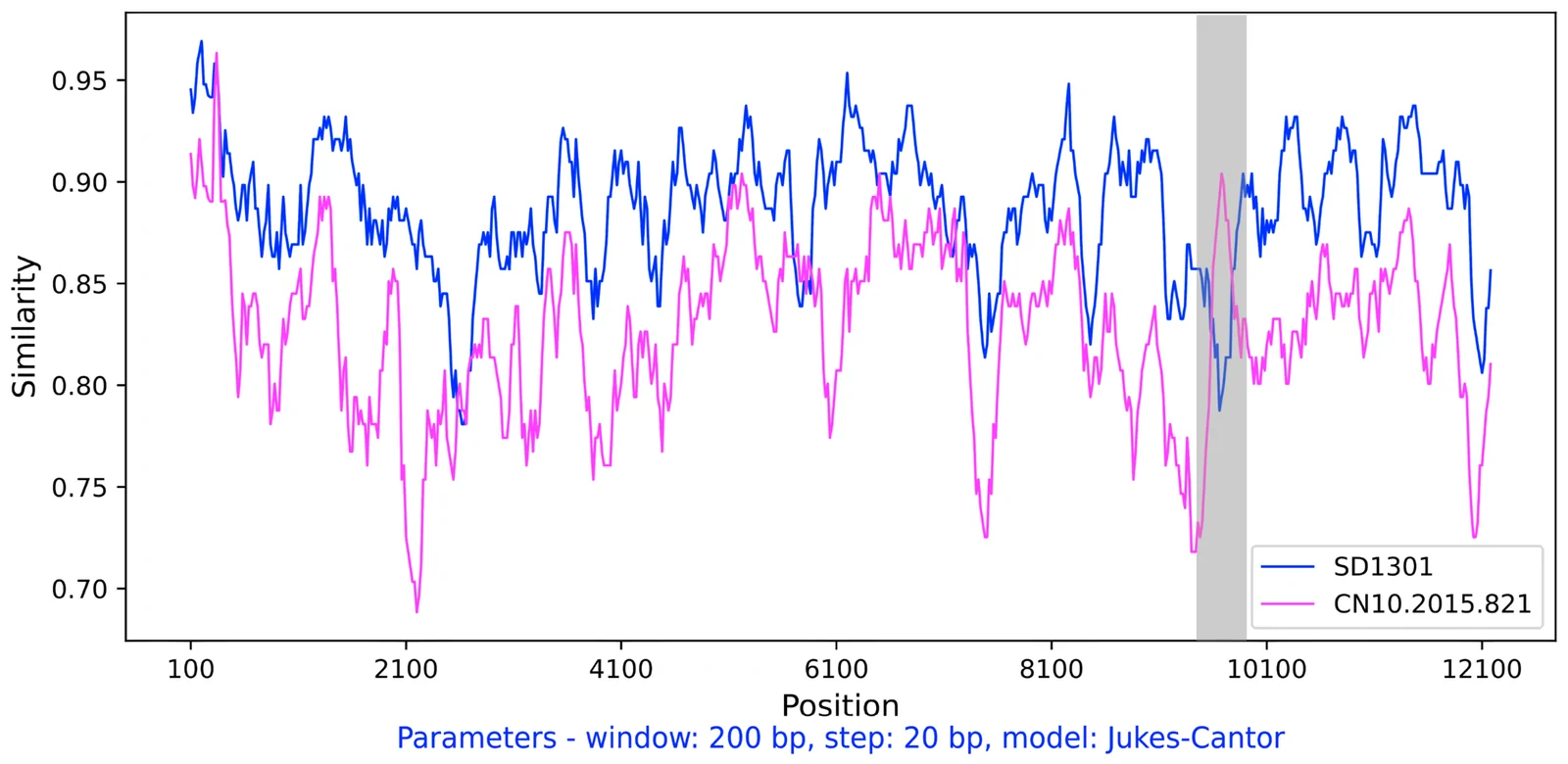
SimPlot analysis of the GX24 genome. The full-length sequence of GX24 served as the query and was scanned against reference sequences SD1301 (blue curve) and CN10.2015.821 (pink curve). The plot was generated using a 200 bp sliding windows and a 20 bp step size under the Jukes–Cantor model. The X-axis represents nucleotide positions, and the Y-axis indicates the percentage of sequence similarity. The gray shaded box highlights the candidate recombinant region (~nt 9583–9762, NS5A gene), where the similarity curve shifts from the major parent (SD1301) to the minor parent (CN10.2015.821), suggesting a putative recombination signal.

A single putative recombination signal was identified in the NS5A gene of GX24 (nt 9583–9762), with the Chinese BVDV-2b isolate SD1301 (KJ000672) and the Italian BVDV-2a isolate CN10.2015.821 (MG879027) inferred as the major and minor parental strains, respectively. This recombination signal was detected by three of the seven algorithms implemented in RDP5 (RDP, BootScan, and SiScan); whereas, GENECONV, MaxChi, Chimaera, and 3Seq did not detect statistically significant evidence of recombination (Table 2). Bayesian phylogenetic analyses further supported this putative recombinant signal. When the candidate recombinant region was excluded, GX24 clustered with the major parental strain SD1301. By contrast, phylogenetic analysis of the putative recombinant region grouped GX24 with the minor parental strain CN10.2015.821 (Figure 3). SimPlot analysis yielded consistent results. Across most of the genome, GX24 exhibited high nucleotide similarity to SD1301 (blue curve). However, within the candidate recombinant region (approximately nt 9583-9762), similarity to SD1301 decreased; whereas, similarity to CN10.2015.821 (pink curve) increased. This shift in sequence similarity is consistent with the presence of a putative recombination breakpoint within the NS5A gene and suggests that this genomic fragment may have originated from a lineage closely related to CN10.2015.821 (Figure 4).

### 3.4. Linear B-Cell Epitopes Prediction of GX24

No signal peptide was predicted in the E2 protein of any of the analyzed strains. DeepTMHMM transmembrane topology prediction indicated that BVDV E2 protein exhibits a typical type I transmembrane architecture, with residues 1–340 forming the ectodomain, residues 340–350 constituting the transmembrane domain (TMD), and residues 350–372 comprising the intracellular domain. Accordingly, only the E2 ectodomain (residues 1–340) was used for all subsequent structural modeling and B-cell epitope analyses to avoid putative interference from the transmembrane and intracellular regions on protein solubility, structural folding, and surface accessibility.

**Figure 5:**
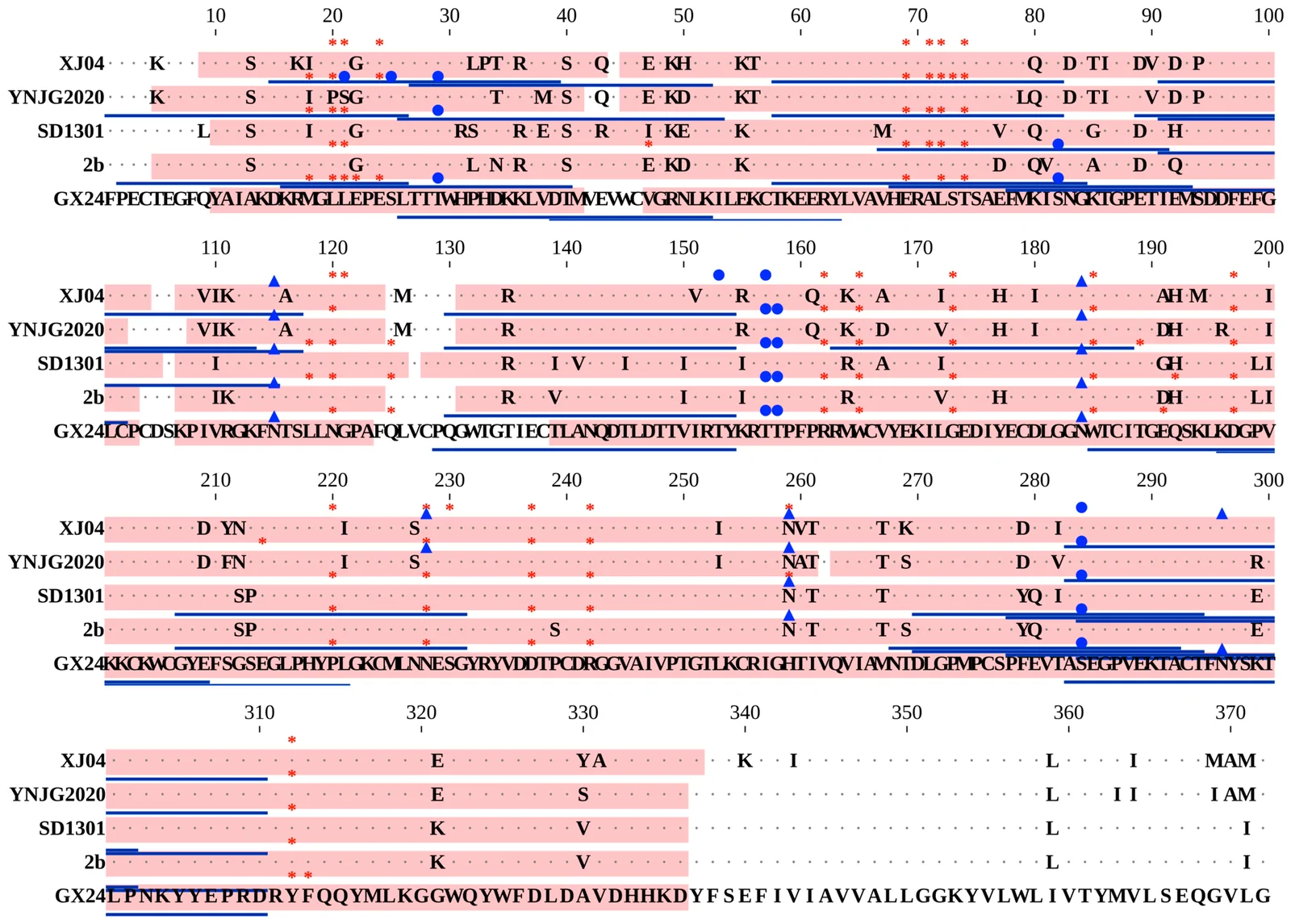
Predicted epitopes and glycosylation sites on the BVDV-2 E2 protein. Multiple sequence alignment of the E2 amino acid sequences from five BVDV-2 strains (i.e., XJ04, YNJG2020, SD1301, 2b, and GX24). Identical residues are denoted by dots (·); whereas, the divergent residues are indicated by single-letter codes. The position numbers are labeled above the alignment at 10-residue intervals. Linear B-cell epitopes predicted by BepiPred-3.0 are highlighted with red translucent backgrounds, and those predicted by Epitope1D are marked in blue underlines, with the overlapping regions staggered vertically to avoid occlusion. Conformational epitope residues predicted by DiscoTope-3.0 are marked with red asterisks (*) above the corresponding amino acid positions. N-linked glycosylation sites predicted by NetNGlyc 1.0 are denoted by blue solid triangles (▲), and O-GalNAc (mucin-type) glycosylation sites predicted by NetOGlyc 4.0 are indicated by blue solid circles (●).

BepiPred-3.0 predicted four major antigenic regions in GX24: R1 (10–41), R2 (47–103), R3 (107–123), and R4 (139–336). The N-terminal region (R1–R3, approximately residues 10–123) was consistently predicted to be highly antigenic across all five strains, although minor differences in epitope boundaries were observed. For instance, the R2 region spanned residues 45–104 in XJ04, 45–102 in YNJG2020, and 47–103 in GX24, indicating the presence of a conserved antigenic hotspot within the N-terminal half of the E2 protein. The C-terminal region (R4) was likewise predicted to be antigenic in all strains, although the starting residue varied slightly, beginning at residue 131 in XJ04 and YNJG2020, residue 128 in SD1301, residue 131 in 2b, and residue 139 in GX24. Collectively, these findings suggest that both the N-terminal and C-terminal regions of the E2 ectodomain contain conserved linear B-cell epitopes across BVDV-2 subgenotypes (Figure 5 and Table S3).

Epitope1D predicted six linear B-cell epitopes in GX24: E1 (26–52), E2 (39–63), E3 (129–154), E4 (185–209), E5 (196–221), and E6 (283–310). In the N-terminal region, the overlapping epitopes (E1 and E2) partially coincided with the BepiPred-3.0 R2 region (47–103). Similarly, E3 (129–154) overlapped with the BepiPred-3.0 R4 region (139–336). In the C-terminal region, E6 (283–310) was consistently predicted in all five strains, suggesting the presence of a highly conserved linear B-cell epitope near the C-terminus. Interestingly, SD1301 and 2b exhibited a greater number of shorter, discontinuous Epitope1D-predicted epitopes in the C-terminal region (e.g., E3–E6 in SD1301 and E6–E11 in 2b); whereas, GX24 and XJ04 contained fewer but longer continuous epitopes. These findings suggest strain-specific differences in the continuity and distribution of predicted linear B-cell epitopes (Figure 5 and Table S3).

### 3.5. Conformational B-Cell Epitopes Prediction of GX24

**Figure 6:**
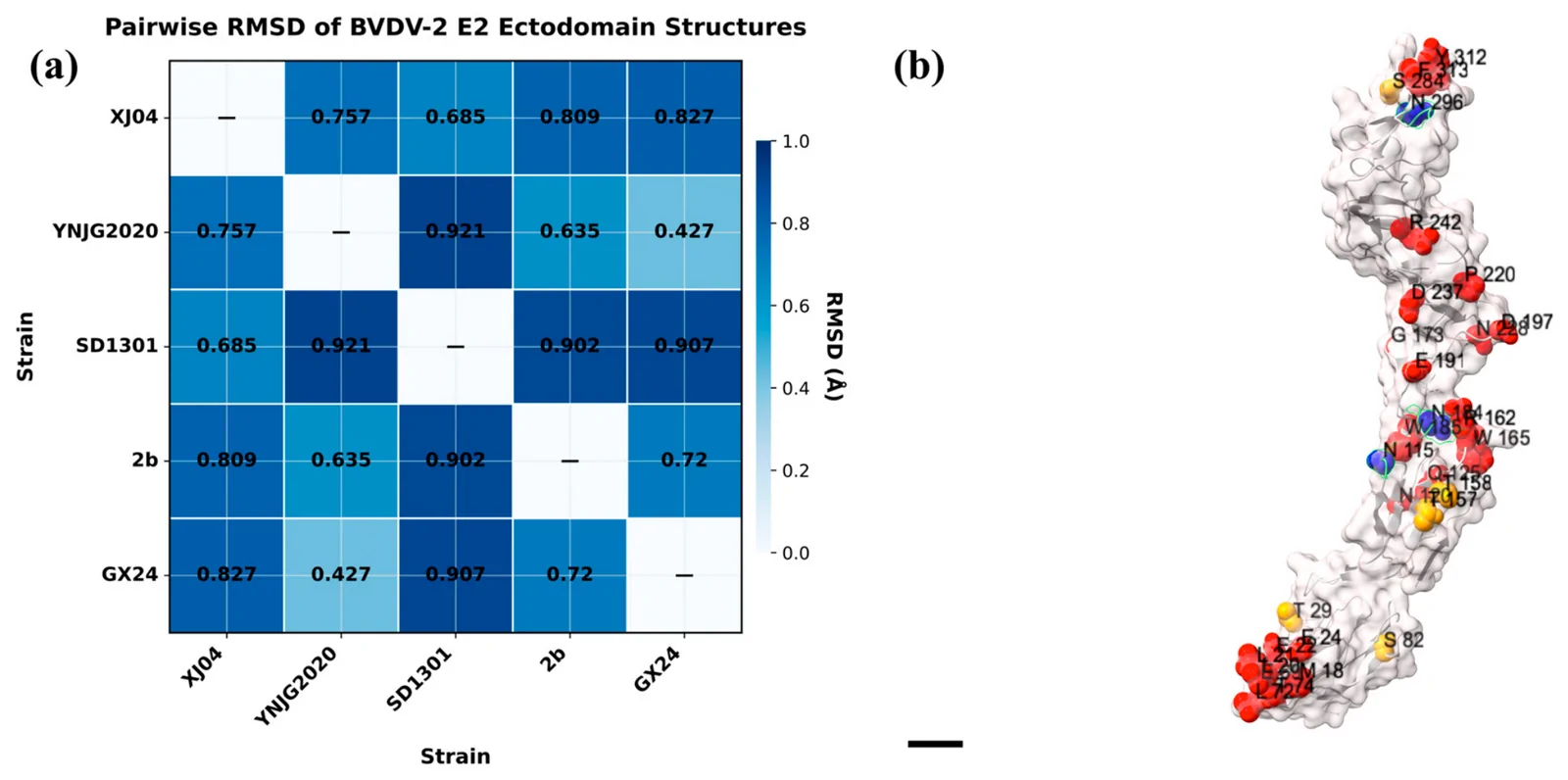
Structural analysis of the BVDV-2 E2 ectodomain. (**a**) Pairwise structural comparison. Heatmap showing root-mean-square deviation (RMSD, Å) values between the predicted structures of five BVDV-2 strains. Diagonal elements (self-comparison) are denoted by “—”. All pairwise RMSD values were <1.0 Å, indicating high structural conservation across the analyzed strains. Color intensity corresponds to the magnitude of RMSD. (**b**) The mapping of glycosylation sites and conformational epitopes on the GX24 E2 ectodomain structure. The structure model (pLDDT ≥ 70) is shown as a gray cartoon, with a 70% transparent surface. N-glycosylation sites are represented by blue spheres, O-GalNAc sites by orange spheres, and predicted epitopes (DiscoTope-3.0 score > 1.5) by red spheres. Scale bar, 10 Å.

The mean pLDDT scores of the five AlphaFold-predicted BVDV-2 E2 ectodomain models ranged from 92.32 to 92.84, with 319–322 residues (93.8–94.7% of the total residues) achieving pLDDT scores ≥70 (Table S4). The removed low-confidence residues (pLDDT <70) were predominantly located at positions 86–88, 286–291, and 329–340 (Table S5), corresponding to flexible loop regions and the membrane-proximal C-terminal segment, which are more difficult to model and are outside the major predicted epitope clusters. After excluding low-confidence residues (18–21 residues per model, 5.3–6.2% of the total), 319–322 high-confidence residues were retained per model for structural comparisons (Table S4). Pairwise structural alignment of the five BVDV-2 E2 ectodomains models revealed a high degree of structural conservation, with RMSD values ranging from 0.427 Å (YNJG2020 vs. GX24) to 0.921 Å (YNJG2020 vs. SD1301), all well below the commonly accepted threshold of 2 Å for structurally similar proteins. These results indicate that the predicted tertiary structures are highly conserved among the analyzed strains (Figure 6a and Table S6). DiscoTope-3.0 predicted 22 surface-exposed residues in GX24 as putative conformational B-cell epitopes residues (Figure 5 and Figure 6b and Table S7). Of these 15 residues (20, 21, 69, 72, 74, 120, 162, 165, 173, 185, 197, 228, 237, 242, and 312) were consistently predicted in all five strains; whereas, residues 71 and 220 were predicted in four of the five strains. These conserved residues likely constitute the core of conformational B-cell epitopes on the E2 protein. In contrast, the absence of residue 71 and the presence of residues 22, 191, and 313 were unique to GX24, suggesting localized structural variation in this newly isolated strain. Additionally, residue 125 was predicted in the three BVDV-2b strains (SD1301, 2b, and GX24). The structural confidence of the predicted epitope regions was further evaluated using local pLDDT scores. The N-terminal epitope cluster (residues 26–103), encompassing both linear (BepiPred-3.0 and Epitope1D) and conformational (DiscoTope-3.0) epitope prediction, exhibited mean pLDDT scores of 91.27–91.99 across the five models. The C-terminal epitope cluster (residues 283–310) showed mean pLDDT scores of 84.29–86.29 (Table S4). Furthermore, the key DiscoTope-3.0-predicted conformational epitope residues at positions 69 (pLDDT: 93.48–95.05), 72 (pLDDT score: 92.64–94.30), and 74 (pLDDT score: 94.11–96.03) all exhibited pLDDT scores ≥70 (Tables S4 and S7).

No C-mannosylation sites were predicted in GX24. By contrast, three N-linked glycosylation sites (N115, N184 and N296) and five O-GalNAc (mucin-type) glycosylation sites (T29, S82, T157, T158 and S284) were predicted (Figure 5). Spatial analysis showed that the N-linked glycosylation site N115 was located within 10 Å of the predicted epitope residues W125 and W185. Moreover, N184 was within 10 Å of residue W165 and W185. In addition, the O-GalNAc sites T29 was located within 10 Å of epitope residue E22. These spatial relationships suggest glycan-mediated masking of B-cell epitopes in GX24.

## 4. Discussion

A previous serological survey reported a BVDV antibody-positive rate of 14.18% (19/134) and identified BVDV-1 as the predominant genotype circulating in Guangxi buffalo herds [23]. In the present study, we reported the isolation and genomic characterization of a BVDV strain obtained from a dairy hybrid buffalo raised under a large-scale intensive farming system in Guangxi Province, China. Phylogenetic analysis classified GX24 within the BVDV-2b subgenotype (Figure 2). Becher et al. (1999) reported that the K2P evolutionary distances among isolates belonging to the same subgenotype was below 14.2% based on the Npro gene [11]. Similarly, our analysis showed that pairwise K2P distances between GX24 and other BVDV-2b isolates based on CNCGs ranged from 12% to 14%. These results are consistent with the phylogenetic assignment of GX24 to subgenotype 2b. However, given the differences in genomic regions, datasets, and evolutionary models, the 14% value should be considered an empirical reference rather than a universal threshold for subgenotype assignment.

Recombination analysis identified a putative recombination signal within the NS5A gene of GX24 (nt 9583–9762; approximately 180 bp), involving a sequence fragment closely related to the Italian BVDV-2a isolate CN10.2015.821 (MG879027) (Table 2, Figure 3 and Figure 4). However, this putative recombination signal was detected by only three of the seven algorithms implemented in RDP5 (RDP, BootScan, and SiScan); whereas, GENECONV, MaxChi, Chimaera, and 3Seq did not identify significant evidence of recombination. Furthermore, the inferred recombinant fragment (~180 bp) approaches the lower sensitivity limit of the sliding-window-based algorithms for recombination implemented in RDP5. Regions slightly smaller than the scanning window size are particularly susceptible to ambiguous signals and increased false-positive rates. Therefore, we describe this finding as a putative recombination signal rather than a confirmed recombination event, and further validation would be needed to confirm this recombination.

The putative recombinant region of GX24 exhibited the highest sequence similarity to the Italian BVDV-2a isolate CN10.2015.821 (MG879027), which represents the closest available reference sequence in current databases. However, this similarity alone does not demonstrate that GX24 originated from Italian buffalo breeding stock. In this study, all publicly available BVDV-2 CNCGs in GenBank were considered, including 18 sequences from China, five from Italy, seven from Japan, and one from South Korea. Nevertheless, complete genome sequences from Southeast Asian countries (including Vietnam, Laos, Thailand, and Myanmar) and neighboring Chinese provinces of Guangxi (such as Yunnan Province and Guizhou Province) remain limited or unavailable. Therefore, we cannot molecularly exclude the possibility that the detected recombinant fragment originated from unsampled local BVDV-2a strains in China or from strains introduced through cross-border livestock trade from Southeast Asia. The geographical proximity between Guangxi Province and Vietnam, coupled with frequent but poorly characterized livestock movement across the China–ASEAN border, underscores the need for targeted molecular surveillance in these under-sampled regions. The historical introduction of Mediterranean buffalo semen and breeding animals from Italy for crossbreeding purposes [58] provides a plausible epidemiological context; notably, the Italian isolate CN10.2015.821 was isolated in 2015, and the introduction of Mediterranean buffalo into Guangxi Province began around 2014. However, additional surveillance and the availability of more geographically representative BVDV-2a genome sequences are needed to determine the actual origin of the recombinant fragment.

The E2 protein is the major envelope glycoprotein of BVDV and plays a critical role in viral entry. It is also the primary target of neutralizing antibodies during BVDV infection [59]. However, the antigenic landscape of E2 remains incompletely characterized. By integrating three computational prediction methods, our bioinformatics analysis predicted both conserved and strain-specific B-cell epitope candidates within the BVDV-2 E2 protein. GX24 shared most predicted core epitopes residues with previously strains, with the N-terminal (26–103) and C-terminal (283–310) regions emerging as the most promising consensus epitope clusters. The N-terminal region (approximately residues 26–103) was consistently predicted by both BepiPred-3.0 and Epitope1D across analyzed strains and contained multiple DiscoTope-3.0 residues (including residues 20, 21, 69, 72, and 74) embedded within this region, indicating a dominant immunogenic cluster (Figure 5). Notably, residues 69–75 encompasses the only known conformational epitope region currently reported of BVDV-2 [60]. The C-terminal region (residues 283–310) was predicted by Epitope1D in all analyzed strains and included DiscoTope-3.0-predicted residue 312, suggesting a conserved linear-conformational composite epitope.

The local pLDDT scores surrounding the predicted epitope regions, including both linear (BepiPred-3.0 and Epitope1D) and conformational (DiscoTope-3.0) predictions, support the reliability of structural comparisons and epitope predictions in these antigenic regions. The N-terminal epitope cluster (residues 26–103) showed high local structural confidence, with mean pLDDT scores ranging from 91.27 to 91.99 across the five models (Table S5). Furthermore, the key DiscoTope-3.0-predicted conformational epitope residues at positions 69, 72, and 74 all achieved pLDDT scores ≥ 70 (93.48–96.03), supporting the structural reliability for conformational epitope prediction (Tables S4 and S7). The C-terminal epitope cluster (residues 283–310) also exhibited high local structural confidence, with mean pLDDT scores ranging from 84.29 to 86.29 (Table S4), well above the confident prediction threshold (pLDDT 70–90) defined by AlphaFold.

To validate our computational predictions, we compared the identified high-confidence epitope regions with experimentally characterized BVDV E2 epitopes reported in previous studies. Although the IEDB does not currently contain BVDV E2-specific linear B-cell epitope data, several independent studies conducted over nearly 30 years (1996–2025) have experimentally mapped epitopes on BVDV E2 epitopes, predominantly conformational epitopes, which provide valuable reference points for positional comparison. Despite the scarcity of experimentally validated BVDV E2 epitope data, Deregt et al. (1998) reported that no epitope mapping studies had involved BVDV-2 [60], and even in the most recent study by Roman-Sosa et al. (2025) demonstrated that all anti-BVDV mAb epitopes were restricted to domain A and were predominantly conformational [61]; the available evidence consistently supports our predicted epitope regions. The N-terminal high-confidence epitope cluster (residues 26–103) encompasses the only experimentally characterized epitope region in BVDV-2 E2 currently reported (residues 69–75 [60]), the type-common epitope of mAb 348 (residues 71–77 [60]), and all anti-BVDV mAb epitopes mapped within domain A [61]. The C-terminal epitope cluster (residues 283–310) is positionally consistent with the *Pestivirus*-conserved YYEP linear epitope located near the C-terminus of E2 protein [62]. Collectively, these comparisons, spanning nearly 30 years of independent experimental studies (1996–2025), support the reliability of our integrative prediction approach while emphasizing the need for experimental validation of the novel epitope candidates identified in this study.

Furthermore, glycosylation of viral envelope proteins has been widely implicated in immune evasion among many enveloped viruses [63,64]. Previous studies have suggested that glycosylation sites located within approximately 10 Å of epitope residues may contribute to glycan shielding and influence antibody accessibility [65,66]. In the present study, spatial analysis of predicted glycosylation sites and identified four conformational B-cell epitope residues (E22, Q125, W165, and W185) that may be affected by glycan shielding. Notably, residue W185 appeared to be influenced by a dual glycan shielding effect from the N-linked glycosylation sites N115 and N184. These findings may inform the design of a novel BVDV-2 vaccine.

Buffalo milk production accounts for 15.27% of global milk production, and China ranks among the world’s third-largest countries in dairy buffalo production [67,68]. In China, Guangxi Province, Yunnan Province, and Guizhou Province represent the main production areas, with Guangxi Province containing the largest dairy buffalo population [58,67]. In southern China, buffalo farming has traditionally been characterized by small family-scale operations using swamp-type buffalo predominantly for draft purposes. Swamp-type buffalo are more tolerant to BVDV infection than riverine-type buffalo [69]. Under this traditional production system, buffalo may have functioned mainly as incidental hosts [70]. However, with the expansion of the dairy buffalo industry, riverine-type buffalo have become increasingly dominant, and intensive large-scale farming systems have replaced traditional family-scale production. These changes in animal breed composition and farming practices may warrant reconsideration of prevention and control strategies for buffalo-associated diarrhea diseases, particularly those caused by BVDV.

BVDV-2 is generally considered more virulent than BVDV-1 and is associated with more severe clinical symptoms [4,5]. In this study, GX24 was isolated from a diarrheic dairy hybrid buffalo maintained in a large-scale intensive farming system. BVDV-2 has been frequently detected in cattle populations managed under large-scale intensive farming or grazing systems in northern and western China [21,32]. To our knowledge, this is the first report of BVDV-2 isolation from dairy buffalo in China. Since commercially available BVDV-1 vaccines do not provide effective protection against BVDV-2 infection [11], and BVDV-2-specific vaccines are currently unavailable in China, greater attention should be directed toward BVDV-2 surveillance and control in Guangxi Province. Notably, the findings of this study are based on a single isolate from one animal, and therefore could not be used to establish the prevalence, circulation, or transmission patterns of BVDV-2b in dairy buffalo populations in Guangxi Province. Further surveillance involving larger sample sizes and broader geographic coverage is required to determine the epidemiological significance of BVDV-2b in buffalo herds in Guangxi Province.

## 5. Conclusions

This study provides the first genomic evidence of a BVDV-2b isolate from dairy buffalo in China, suggesting that BVDV-2b is present in this population rather than establishing its circulation or prevalence. The isolate GX24 presents a putative recombination signal in the NS5A gene; however, its epidemiological origin remains uncertain as the availability of BVDV-2 CNCGs from Guangxi Province and adjacent regions is limited. The integrative bioinformatics analysis of the E2 protein predicted conserved N-terminal and C-terminal B-cell epitope clusters that represent putative targets for vaccine development. Notably, several predicted epitope residues may be affected by glycosylation, which should be considered during antigen design. Given the continued expansion of the dairy buffalo industry in Guangxi Province and the current lack of BVDV-2 vaccines in China, further molecular surveillance is required to determine the prevalence and distribution of BVDV-2b in dairy buffalo populations in China and suggest that appropriate measures should be considered to prevent and control the disease.

## Figures and Tables

**Table 1:** Genome structure of the BVDV-2 isolate GX24.

Gene	Begin Site	End Site	Length(nt)
5′ UTR	1	371	371
N^pro^	372	875	504
C	876	1181	306
E^rns^	1182	1862	681
E1	1863	2447	585
E2	2448	3563	1116
P7	3564	3773	210
NS2	3774	5132	1359
NS3	5133	7181	2049
NS4A	7182	7373	192
NS4B	7374	8414	1041
NS5A	8415	9905	1491
NS5B	9906	12,06 5	2160
3′ UTR	12,066	12,267	202

**Table 2:** Putative recombination signals identified in the isolate GX24.

Recombinant	Minor Parental Sequence (BVDV-2a)	Major Parental Sequence (BVDV-2b)	Breakpoint Begin (nt)	Breakpoint End (nt)	RDP *p*-Value (Corr.)	Geneconv *p*-Value	BootScan *p*-Value (Corr.)	MaxChi *p*-Value	Chimaera *p*-Value	SiScan *p*-Value (Corr.)	3Seq *p*-Value
GX24 (PX682047, BVDV-2b, China)	CN10.2015.821 (MG879027, BVDV-2a, Italy)	SD1301 (KJ000672, BVDV-2b, China)	9583	9762	9.04 × 10^−4^ (9.04 × 10^−3^)	NS	1.13 × 10^−3^ (1.13 × 10^−2^)	NS	NS	2.07 × 10^−7^ (2.07 × 10^−6^)	NS

NS = No significant recombination signal was detected by the method in question. Uncorrected *p*-values were obtained directly from the RDP5 output; whereas, Bonferroni-corrected *p*-values (shown in parentheses) were calculated by multiplying each uncorrected *p*-value by the total number of recombination signals detected in the analysis (N = 10). All three significant corrected *p*-values remained below 0.05, indicating that the putative recombination signal remained statistically significant after correction for multiple testing.

## Data Availability

The original contributions presented in this study are included in the article/Supplementary Material. Further inquiries can be directed to the corresponding authors.

## Supplementary Materials

The following supporting information can be downloaded at: https://www.mdpi.com/article/10.3390/microorganisms14081845/s1, Figure S1: Detection of BVDV RNA and virions in GX24-inoculated MDBK cell cultures; Table S1: Summary of sequences analyzed in this study; Table S2. Pairwise nucleotide sequence identity (%) among BVDV-1, BVDV-2, BVDV-3, and other Pestivirus isolates based on near-complete coding genome sequences; Table S3: Prediction linear B-cell epitopes in the BVDV-2 E2 amino acid sequence using BepiPred-3.0 and Epitope1D; Table S4: pLDDT confidence scores and local structural confidence around predicted epitope regions for five AlphaFold-predicted BVDV-2 E2 ectodomain models; Table S5. pLDDT scores of low-confidence residues removed prior to structural alignment; Table S6: Pairwise structural alignment details for five AlphaFold-predicted BVDV-2 E2 ectodomain models; Table S7: Residues of the BVDV E2 ectodomain predicted as conformational B-cell epitopes.

## Abbreviations

The following abbreviations are used in this manuscript:

BVDV	Bovine viral diarrhea virus
BRDC	Bovine respiratory disease complex
CNCGs	Complete or near-complete genomes
CP	Cytopathic
CPE	Cytopathic effects
DAPI	4′,6-Diamidino-2-phenylindole
FBS	Fetal bovine serum
FITC	Fluorescein isothiocyanate
GTR	General Time Reversible
IFA	Immunofluorescence assay
K2P	Kimura 2-parameter
mAb	Monoclonal antibody
MCMC	Markov chain Monte Carlo
MDBK	Madin-Darby bovine kidney
MEM	Minimum Essential Medium
ML	Maximum likelihood
NCD	Neonatal calf diarrhea
NCP	Non-cytopathic
nt	Nucleotide(s)
ORF	Open reading frame
PI	Persistently infected
pLDDT	Predicted local distance difference test
PVDF	Polyvinylidene difluoride
RDP	Recombination Detection Program
RMSD	Root-mean-square deviation
RT-PCR	Reverse transcription polymerase chain reaction
TEM	Transmission electron microscopy
TMD	Transmembrane domain
UTR	Untranslated region
