# Supplementary material for: Isolation and Genomic Characteristics of the First Bovine Viral Diarrhea Virus Subgenotype 2b Isolate from Buffalo in Guangxi Province, China
Source: Microorganisms. 2026 Aug 19;14(8):1845. doi: 10.3390/microorganisms14081845 (PMC13515615; doi:10.3390/microorganisms14081845)
Supplement: Supplementary file 1 [file microorganisms-14-01845-s001.zip › Figure S1.pdf]

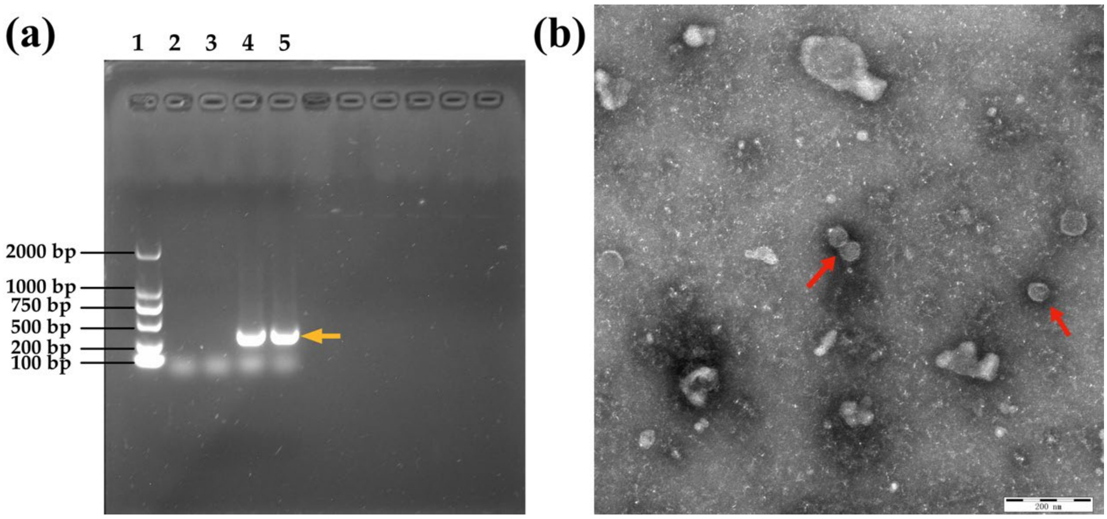

Figure S1. Detection of BVDV RNA and virions in GX24-inoculated MDBK cell cultures. (a) RT-PCR detection of BVDV RNA. Lane 1, DL2000 DNA marker (Takara, Japan); lane 2, no-template control (NTC); lane 3, uninfected MDBK cells (negative control); lane 4, BVDV NADL strain (positive control); lane 5, GX24 isolate. The arrow indicates the expected 283-bp amplicon. (b) Negative-stain transmission electron micrograph of the culture supernatant showing spherical virions approximately 50 nm in diameter, morphologically consistent with members of the genus *Pestivirus*. Arrows indicate representative virions. Scale bar, 200 nm.
