# Supplementary material for: Isolation and Genomic Characteristics of the First Bovine Viral Diarrhea Virus Subgenotype 2b Isolate from Buffalo in Guangxi Province, China
Source: Microorganisms. 2026 Aug 19;14(8):1845. doi: 10.3390/microorganisms14081845 (PMC13515615; doi:10.3390/microorganisms14081845)
Supplement: Supplementary file 1 [file microorganisms-14-01845-s001.zip › Table S1.pdf]

Table S1. Summary of sequences analyzed in this study

GenBank Number	Strain	Country	Year	Species	Subgenotype
AB078950	KS86-1ncp	Japan	1986	Pestivirus bovis(BVDV-1)	1j
AB567658	Hokudai-Lab/09	Japan	2009	Pestivirus tauri(BVDV-2)	2b
AF002227	C413	United States	NA	Pestivirus tauri(BVDV-2)	2a
AF041040	C24V	United States	1959	Pestivirus bovis(BVDV-1)	1a
AF144617	H138	Kenya	1967	Pestivirus giraffae	-
AF502399	New York 93	United States	1993	Pestivirus tauri(BVDV-2)	2a
AF526381	ZM-95	China	1995	Pestivirus bovis(BVDV-1)	1m
AY149215	p11Q	Canada	NA	Pestivirus tauri(BVDV-2)	2a
AY149216	p24515	Canada	NA	Pestivirus tauri(BVDV-2)	2a
DQ088995	Singer	United States	1974	Pestivirus bovis(BVDV-1)	1a
FJ040215	Th/04KhonKaen	Thailand	2004	Pestivirus brazilense(BVDV-3)	3a
FJ527854	XJ-04	China	2004	Pestivirus tauri(BVDV-2)	2a
GQ888686	JZ05-1	China	2005	Pestivirus tauri(BVDV-2)	2a
HG426491	Potsdam1600	Germany	2000	Pestivirus tauri(BVDV-2)	2a
HG426493	SH2210-17	Germany	2010	Pestivirus tauri(BVDV-2)	2a
HG426495	VOE4407	Germany	2007	Pestivirus tauri(BVDV-2)	2a
HQ258810	SH-28	China	2009	Pestivirus tauri(BVDV-2)	2a
JF714967	HLJ-10	China	2011	Pestivirus tauri(BVDV-2)	2a
JN400273	SD0803	China	2008	Pestivirus bovis(BVDV-1)	1q
JQ799141	M31182	China	2010	Pestivirus bovis(BVDV-1)	1u
JX428945	Aydin04-TR	Turkey	2004	Pestivirus aydinense	-
KC695810	camel-6	China	2010	Pestivirus bovis(BVDV-1)	1q
KC695814	Av69VEDEVAC	Hungary	1973	Pestivirus bovis(BVDV-1)	1b
KC757383	10JJ-SKR	Korea	2010	Pestivirus bovis(BVDV-1)	1d
KC853441	SuwaCp	Switzerland	1993	Pestivirus bovis(BVDV-1)	1k
KC963968	11F011	Korea	2011	Pestivirus tauri(BVDV-2)	2a
KF896608	Bega-like	Australia	2012	Pestivirus bovis(BVDV-1)	1c
KJ000672	SD1301	China	2012	Pestivirus tauri(BVDV-2)	2b
KP313732	Carlito	Switzerland	2014	Pestivirus bovis(BVDV-1)	1e
KP941582	USMARC-53873	United States	2014	Pestivirus tauri(BVDV-2)	2a
KT832818	USMARC-60765	United States	2014	Pestivirus tauri(BVDV-2)	2a
KT832821	USMARC-60768	United States	2014	Pestivirus tauri(BVDV-2)	2a
KT832822	USMARC-60779	United States	2014	Pestivirus tauri(BVDV-2)	2a
KT832823	USMARC-60780	United States	2014	Pestivirus tauri(BVDV-2)	2a
KU563155	HN1507	China	2015	Pestivirus brazilense(BVDV-3)	3a
KX096718	HB-1511	China	2015	Pestivirus tauri(BVDV-2)	2a
KX577637	SLO/2407/2006	Slovenia	2006	Pestivirus bovis(BVDV-1)	1e
KX857724	ACM/BR/2016	Brazil	2016	Pestivirus bovis(BVDV-1)	1i
KX987157	SLO/1170/2000	Slovenia	2000	Pestivirus bovis(BVDV-1)	1f

LC089875	IS26/01ncp	Japan	2001	Pestivirus bovis(BVDV-1)	1o
LC089876	Shitara/02/06	Japan	2006	Pestivirus bovis(BVDV-1)	1n
LC649064	KZ-91-NCP	Japan	1991	Pestivirus tauri(BVDV-2)	2a
LC727649	Tokachi-47	Japan	2017	Pestivirus tauri(BVDV-2)	2a
LC727650	Tokachi-31	Japan	2016	Pestivirus tauri(BVDV-2)	2a
LT631725	UM/126/07	Italy	2007	Pestivirus bovis(BVDV-1)	1h
LT837585	VE24512	Italy	2012	Pestivirus bovis(BVDV-1)	1r
M31182	NADL	United States	1963	Pestivirus bovis(BVDV-1)	1a
M96687	Osloss	Belgium	1967	Pestivirus bovis(BVDV-1)	1b
M96751	1-SD1	United States	1992	Pestivirus bovis(BVDV-1)	1a
MG879027	CN10.2015.821	Italy	2014	Pestivirus tauri(BVDV-2)	2a
MG923683	LV01-2018	Brazil	2015	Pestivirus bovis(BVDV-1)	1d
MH231124	1786c	United States	1989	Pestivirus tauri(BVDV-2)	2e
MH231132	AzSpl	United States	1997	Pestivirus tauri(BVDV-2)	2a
MH231145	Victor301	United States	1990	Pestivirus tauri(BVDV-2)	2a
MH231148	12-149150	United States	2012	Pestivirus tauri(BVDV-2)	2e
MH231149	Short	United States	1989	Pestivirus tauri(BVDV-2)	2c
MH231150	12-151955-317	United States	2012	Pestivirus tauri(BVDV-2)	2e
MH231151	14622	United States	2005	Pestivirus tauri(BVDV-2)	2c
MH231152	2412	United States	1989	Pestivirus tauri(BVDV-2)	2c
MH806434	125c	United States	1990	Pestivirus tauri(BVDV-2)	2a
MH806435	1336H	United States	2005	Pestivirus tauri(BVDV-2)	2a
MH806436	296c	United States	1995	Pestivirus tauri(BVDV-2)	2a
MH806437	9231	United States	2004	Pestivirus tauri(BVDV-2)	2a
MH806438	McCart c	United States	1989	Pestivirus tauri(BVDV-2)	2a
MK599227	SD-1	China	2016	Pestivirus tauri(BVDV-2)	2a
MN527354	GS2018	China	2018	Pestivirus tauri(BVDV-2)	2a
MW006485	HEN01	China	2014	Pestivirus tauri(BVDV-2)	2b
MW054936	UM/111/06	Italy	2006	Pestivirus bovis(BVDV-1)	1g
MW168422	YNJG2020	China	2020	Pestivirus tauri(BVDV-2)	2a
MW528234	D66-11-28	Germany	2011	Pestivirus tauri(BVDV-2)	2a
MW528235	TV02-13T136	Germany	2013	Pestivirus tauri(BVDV-2)	2a
ON676188	Mungyeong	Korea	2019	Pestivirus tauri(BVDV-2)	2a
OP792028	BVDV2NM21	China	2021	Pestivirustauri(BVDV-2)	2b
OQ338174	22Anhui-7	China	2022	Pestivirus tauri(BVDV-2)	2a
OQ338175	22Gansu-F2	China	2022	Pestivirus tauri(BVDV-2)	2a
OQ338176	22Gansu-F3	China	2022	Pestivirus tauri(BVDV-2)	2a
OQ338177	22Sichuan-B8	China	2022	Pestivirus tauri(BVDV-2)	2a
OQ411019	Ind/TN-1214/19	India	2019	Pestivirus brazilense(BVDV-3)	3e
OQ411020	Ind/ABI15385/12	India	2012	Pestivirus brazilense(BVDV-3)	3c
OQ411021	Ind/BHA5309/12	India	2012	Pestivirus brazilense(BVDV-3)	3d
PP823882	BVDV-2/JS-2/2023	China	2023	Pestivirus tauri(BVDV-2)	2b
PP854293	2b	China	2023	Pestivirus tauri(BVDV-2)	2b

PV541660	BVDV2OV853	United States	2018	Pestivirus tauri(BVDV-2)	2a
PV541661	BVDV2OV856	United States	2018	Pestivirus tauri(BVDV-2)	2a
PX121309	IT/2005206041	Italy	2005	Pestivirus tauri(BVDV-2)	2a
PX121310	IT/2015277457-2	Italy	2015	Pestivirus tauri(BVDV-2)	2a
PX121311	IT/2016169121	Italy	2016	Pestivirus tauri(BVDV-2)	2a
PX121312	IT/201864643	Italy	2018	Pestivirus tauri(BVDV-2)	2a
PX122060	277	United States	1993	Pestivirus tauri(BVDV-2)	2a
PX122061	2251	United States	NA	Pestivirus tauri(BVDV-2)	2a
PX122062	2541	United States	NA	Pestivirus tauri(BVDV-2)	2a
PX122063	3590	United States	1989	Pestivirus tauri(BVDV-2)	2a
PX122064	5111103	United States	2005	Pestivirus tauri(BVDV-2)	2a
PX122065	7092c	United States	1989	Pestivirus tauri(BVDV-2)	2a
PX682047	GX24	China	2024	Pestivirus tauri(BVDV-2)	2b
U18059	890	United States	1990	Pestivirus tauri(BVDV-2)	2a
U63479	CP7	United States	1985	Pestivirus bovis(BVDV-1)	1b

NA: not available, “-”: not belongs the BVDV group.
