# Supplementary material for: Isolation and Genomic Characteristics of the First Bovine Viral Diarrhea Virus Subgenotype 2b Isolate from Buffalo in Guangxi Province, China
Source: Microorganisms. 2026 Aug 19;14(8):1845. doi: 10.3390/microorganisms14081845 (PMC13515615; doi:10.3390/microorganisms14081845)
Supplement: Supplementary file 1 [file microorganisms-14-01845-s001.zip › Table S3.pdf]

Table S3. Prediction linear B-cell epitopes in the BVDV-2 E2 amino acid sequence using BepiPred-3.0 and Epitope1D.

Strain	BepiPred-3.0		Epitope1D		Overlap		
	Region	Range	Region	Range	Range	Length (aa)	Sequence
XJ04	R1	9-43	E1	15-39	15-39	25	DKKIGLLGPESLTTTWHLPTKRLVD
	R1	9-43	E2	27-52	27-43	17	TTTWHLPTKRLVDSMVQ
	R2	45-104	E2	27-52	45-52	8	WCEGKHLK
	R2	45-104	E3	58-82	58-82	25	TKEERYLVAVHERALSTSAEFMQIS
	R2	45-104	E4	91-117	91-104	14	IDMPDDFEFGLCPC
	R3	107-124	E4	91-117	107-117	11	KPVIKGKFNAS
	R4	131-337	E5	130-154	131-154	24	GWTGRIECTLANQDTLDTTVVRTY
	R4	131-337	E6	283-310	283-310	28	ASEGPVEKTACTFNYSKTLPNKYYEPRD
YNJG2020	R1	5-41	E1	1-26	5-26	22	KEGFQYAISKDKRIGPSGPESL
	R1	5-41	E2	26-53	26-41	16	LTTTWHPHTKKLMDSM
	R2	45-102	E2	26-53	45-53	9	WCEGKDLKI
	R2	45-102	E3	58-82	58-82	25	TKEERYLVAVHERALSTSAEFLQIS
	R2	45-102	E4	89-113	89-102	14	EVIDMPDDFEFGLC
	R2	45-102	E5	91-117	91-102	12	IDMPDDFEFGLC
	R3	108-124	E4	89-113	108-113	6	PVIKGK
	R3	108-124	E5	91-117	108-117	10	PVIKGKFNAS
	R4	131-261	E6	130-154	131-154	24	GWTGRIECTLANQDTLDTTVIRTY
	R4	131-261	E7	163-188	163-188	26	RKWCDYEKIVGEDIHECILGGNWTCI
	R5	263-336	E8	283-310	283-310	28	ASEGPVEKTACTFNYSRTLPNKYYEPRD
SD1301	R1	10-105	E1	67-91	67-91	25	MHERALSTSAVFMQISNGGTGPDTI
	R1	10-105	E2	91-115	91-105	15	IHMSDDFEFGLCPCD
	R2	107-126	E2	91-115	107-115	9	KPIIRGKFN

	R3	128-336	E3	207-231	207-231	25	GYEFSSPEGLPHYPLGKCMLNNESG
	R3	128-336	E4	270-294	270-294	25	DLGPMPCSPYQVIASEGPVEKTACT
	R3	128-336	E5	278-302	278-302	25	PYQVIASEGPVEKTACTFNYSETLP
	R3	128-336	E6	284-310	284-310	27	SEGPVEKTACTFNYSETLPNKYYEPRD
2b	R1	5-103	E1	2-26	5-26	22	TEGFQYAISKDKRMGLLGPESL
	R1	5-103	E2	16-40	16-40	25	KRMGLLGPESLTTTWHLHNKRLVDS
	R1	5-103	E3	58-84	58-84	27	TKEERYLVAVHERALSTSADFMQVSNG
	R1	5-103	E4	68-93	68-93	26	HERALSTSADFMQVSNGATGPDTIQM
	R1	5-103	E5	78-102	78-102	25	FMQVSNGATGPDTIQMSDDFEFGLC
	R2	107-124	-	-	-	-	-
	R3	131-336	E6	130-154	131-154	24	GWTGRIECVLANQDTLDTTIIRTY
	R3	131-336	E7	207-231	207-231	25	GYEFSSPEGLPHYPLGKCMLNNESG
	R3	131-336	E8	268-292	268-292	25	NSDLGPMPCSPYQVTASEGPVEKTA
	R3	131-336	E9	270-294	270-294	25	DLGPMPCSPYQVTASEGPVEKTACT
	R3	131-336	E10	278-302	278-302	25	PYQVTASEGPVEKTACTFNYSETLP
	R3	131-336	E11	284-310	284-310	27	SEGPVEKTACTFNYSETLPNKYYEPRD
GX24	R1	10-41	E1	26-52	26-41	16	LTTTWHPHDKKLVDTM
	R1	10-41	E2	39-63	39-41	3	DTM
	R2	47-103	E1	26-52	47-52	6	VGRNLK
	R2	47-103	E2	39-63	47-63	17	VGRNLKILEKCTKEERY
	R3	107-123	-	-	-	-	-
	R4	139-336	E3	129-154	139-154	16	TLANQDTLDTTVIRTY
	R4	139-336	E4	185-209	185-209	25	WTCITGEQSKLKDGPVKKCKWCGYE
	R4	139-336	E5	196-221	196-221	26	KDGPVKKCKWCGYEFSGSEGLPHYPL
	R4	139-336	E6	283-310	283-310	28	ASEGPVEKTACTFNYSKTLPNKYYEPRD

Predicted epitope intervals from BepiPred-3.0 (R) and Epitope1D (E) are listed per strain. Overlapping regions were determined by coordinate intersection, with the consensus sequence and length

(aa) shown. Dashes (–) indicate non-overlapping intervals. For BepiPred-3.0, adjacent fragments from the same strain were merged; for Epitope1D, only consecutive 25-mer windows (Prediction = 1) were merged. GX24 denotes the novel BVDV-2b clinical isolate; XJ04, SD1301, YNJG2020, and 2b are reference strains.
