# Supplementary material for: Isolation and Genomic Characteristics of the First Bovine Viral Diarrhea Virus Subgenotype 2b Isolate from Buffalo in Guangxi Province, China
Source: Microorganisms. 2026 Aug 19;14(8):1845. doi: 10.3390/microorganisms14081845 (PMC13515615; doi:10.3390/microorganisms14081845)
Supplement: Supplementary file 1 [file microorganisms-14-01845-s001.zip › Table S4.pdf]

Table S4. pLDDT confidence scores and local structural confidence around predicted epitope regions for five AlphaFold-predicted BVDV-2 E2 ectodomain models. All pLDDT values are represented by Cα atom values; whole residues with Cα pLDDT <70 were removed prior to structural comparison.

Parameter	2b	GX24	SD1301	XJ04	YNJG2020
Total residues (original)	340	340	340	340	340
Mean pLDDT	92.78	92.65	92.84	92.32	92.35
Residues with pLDDT ≥70	321	319	322	320	320
Residues with pLDDT <70	19	21	18	20	20
Residues retained after removal	321	319	322	320	320
N-terminal cluster (26–103) mean pLDDT	91.99	91.94	91.98	91.27	91.47
C-terminal cluster (283–310) mean pLDDT	85.53	84.29	86.29	85.26	84.34
Residue 69 pLDDT	95.05	95.38	94.68	93.96	93.48
Residue 72 pLDDT	94.30	93.03	93.42	93.30	92.64
Residue 74 pLDDT	96.03	94.11	95.61	95.34	94.84
