# Supplementary material for: Isolation and Genomic Characteristics of the First Bovine Viral Diarrhea Virus Subgenotype 2b Isolate from Buffalo in Guangxi Province, China
Source: Microorganisms. 2026 Aug 19;14(8):1845. doi: 10.3390/microorganisms14081845 (PMC13515615; doi:10.3390/microorganisms14081845)
Supplement: Supplementary file 1 [file microorganisms-14-01845-s001.zip › Table S5.pdf]

Table S5. pLDDT values of low-confidence residues removed prior to structural alignment.

Strain	Removed residues	n	Mean pLDDT	pLDDT range
2b	86–87	2	63.39	61.45–65.34
2b	286–291	6	65.56	62.40–68.48
2b	330–340	11	63.33	54.86–68.87
GX24	86–88	3	64.12	60.28–68.55
GX24	286–291	6	62.25	58.73–65.96
GX24	329–340	12	62.24	53.58–69.35
SD1301	86–87	2	63.59	60.65–66.53
SD1301	287–291	5	67.45	66.03–69.04
SD1301	330–340	11	63.45	54.89–68.75
XJ04	86–88	3	65.10	63.88–67.26
XJ04	286–291	6	66.54	64.64–69.09
XJ04	330–340	11	62.91	52.96–69.18
YNJG2020	86–87	2	66.18	65.49–66.87
YNJG2020	286–292	7	63.47	59.33–69.66
YNJG2020	330–340	11	61.40	52.26–67.15

Whole residues with Cα pLDDT <70 were removed from each AlphaFold3-predicted BVDV-2 E2 ectodomain model (2b: 19; GX24: 21; SD1301: 18; XJ04: 20; YNJG2020: 20) before pairwise superposition with ChimeraX Matchmaker (see Table S4). Listed regions are contiguous removed residue ranges; mean and range refer to Cα atom pLDDT values within each region.
