# Supplementary material for: Isolation and Genomic Characteristics of the First Bovine Viral Diarrhea Virus Subgenotype 2b Isolate from Buffalo in Guangxi Province, China
Source: Microorganisms. 2026 Aug 19;14(8):1845. doi: 10.3390/microorganisms14081845 (PMC13515615; doi:10.3390/microorganisms14081845)
Supplement: Supplementary file 1 [file microorganisms-14-01845-s001.zip › Table S6.pdf]

Table S6. Pairwise structural alignment details for five AlphaFold-predicted BVDV-2 E2 ectodomain models.

Comparison	Pruned atom pairs	Pruned RMSD (Å)	All atom pairs	All pairs RMSD (Å)
2b vs GX24	305	0.720	319	0.869
2b vs SD1301	310	0.902	321	0.993
2b vs XJ04	272	0.809	320	1.374
2b vs YNJG2020	318	0.635	320	0.770
GX24 vs SD1301	308	0.907	319	0.992
GX24 vs XJ04	310	0.827	319	0.913
GX24 vs YNJG2020	317	0.427	318	0.442
SD1301 vs XJ04	239	0.685	320	2.061
SD1301 vs YNJG2020	316	0.921	320	1.024
XJ04 vs YNJG2020	298	0.757	319	0.996
