# Supplementary material for: Isolation and Genomic Characteristics of the First Bovine Viral Diarrhea Virus Subgenotype 2b Isolate from Buffalo in Guangxi Province, China
Source: Microorganisms. 2026 Aug 19;14(8):1845. doi: 10.3390/microorganisms14081845 (PMC13515615; doi:10.3390/microorganisms14081845)
Supplement: Supplementary file 1 [file microorganisms-14-01845-s001.zip › Table S7.pdf]

Table S7. Residues of the BVDV E2 ectodomain predicted as conformational B-cell epitopes

Strian	Residue Number	Residue	Discotope Score	pLDDT
XJ04	20	L	3.0963	94.98
	21	L	1.80824	93.80
	24	E	1.53931	93.89
	69	E	1.745	93.96
	71	A	1.6638	94.18
	72	L	2.13094	93.30
	74	T	1.80441	95.34
	120	N	1.87568	95.76
	121	G	1.77817	94.71
	162	R	2.74936	96.53
	165	W	2.83421	95.01
	173	G	2.17186	95.52
	185	W	3.08291	96.53
	197	D	2.15555	94.47
	220	P	1.54888	97.55
	228	N	1.64183	95.36
	230	S	1.59435	95.40
	237	D	2.13176	96.28
	242	R	1.76751	95.64
	259	N	1.58333	96.17
	312	Y	2.16721	85.48
YNJG2020	18	I	1.63766	95.65
	20	P	3.5173	94.93
	21	S	1.63546	93.59
	24	E	1.52974	93.65
	69	E	2.98337	93.48
	71	A	1.66645	93.37
	72	L	1.96629	92.64
	73	S	1.59878	92.20
	74	T	1.53294	94.84
	120	N	2.09366	96.09
	162	R	2.28007	96.94
	165	W	2.50133	95.73
	173	G	2.15738	95.90
	185	W	2.82593	96.80
	197	D	2.07999	95.15
	214	E	1.76502	96.01
	228	N	1.75677	95.65
	237	D	2.67894	96.47
	242	R	2.16417	95.93

	312	Y	1.94374	86.37
SD1301	18	I	1.54159	96.46
	20	L	3.12857	95.26
	21	L	2.06076	94.31
	69	E	2.06696	94.68
	71	A	1.61521	94.17
	72	L	2.09597	93.42
	74	T	1.52973	95.61
	118	L	1.57425	96.73
	120	N	2.48305	96.17
	125	Q	1.86672	97.60
	162	R	2.49026	97.02
	165	W	2.51617	95.53
	173	G	2.34119	96.12
	185	W	2.90114	96.73
	189	T	1.54916	97.18
	197	D	2.03722	94.99
	220	P	1.88104	97.56
	228	N	1.92638	95.05
	237	D	2.37294	96.66
	242	R	1.90175	96.21
	259	N	1.61886	96.66
	312	Y	1.67542	87.16
2b	20	L	3.15883	95.85
	21	L	1.95979	94.63
	47	E	1.57163	93.52
	69	E	2.29554	95.05
	71	A	1.5998	95.19
	72	L	2.0319	94.30
	74	T	1.67382	96.03
	118	L	1.69434	96.94
	120	N	2.24075	96.27
	125	Q	2.08714	97.71
	162	R	2.62709	97.10
	165	W	2.35187	95.70
	173	G	2.25543	96.00
	185	W	3.25546	96.90
	192	H	1.66179	97.06
	197	D	2.14822	94.69
	220	P	1.92679	97.48
	228	N	1.9618	95.06
	237	D	2.32425	96.48
	242	R	1.68586	96.21

	312	Y	2.02142	86.47
GX24	18	M	2.09107	94.97
	20	L	3.09897	93.87
	21	L	2.16743	91.87
	22	E	1.74103	92.71
	24	E	1.99863	91.19
	69	E	2.26197	95.38
	72	L	2.0728	93.03
	74	T	1.68047	94.11
	120	N	2.28207	96.58
	125	Q	1.71646	98.03
	162	R	2.79616	97.22
	165	W	2.70482	95.91
	173	G	1.98328	96.65
	185	W	2.86321	97.01
	191	E	1.55451	96.96
	197	D	2.16944	94.95
	220	P	1.77264	97.57
	228	N	1.91167	95.17
	237	D	2.28728	97.17
	242	R	1.92007	96.51
	312	Y	2.03453	85.97
	313	F	1.71865	88.90

Selected residues with DiscoTope-3.0 scores > 1.5 and predicted Local Distance Difference Test (pLDDT) values > 70 are listed. GX24 represents the newly isolated BVDV-2b clinical strain (2024), whereas XJ04, YNJG2020, SD1301, and 2b are reference strains of BVDV-2a and BVDV-2b subtypes. Residue numbering corresponds to the ectodomain sequence of each strain. DiscoTope-3.0 score indicates the predicted epitope propensity, and pLDDT reflects the local structural confidence from AlphaFold3 modeling (Cα atom values).
